# Using a Virtual Reality Social Network During Awake Craniotomy to Map Social Cognition: Prospective Trial

**DOI:** 10.2196/10332

**Published:** 2018-06-26

**Authors:** Florian Bernard, Jean-Michel Lemée, Ghislaine Aubin, Aram Ter Minassian, Philippe Menei

**Affiliations:** ^1^ Neurosurgery CHU Angers Angers France; ^2^ Laboratoire d’Anatomie Faculté de Médecine d’Angers Angers France; ^3^ CRCINA, INSERM Université de Nantes, Université d'Angers Angers France; ^4^ CHU Angers Département d’Ortophonie Angers France; ^5^ CHU Angers Département d’Anesthésie-Réanimation Angers France; ^6^ CHU Angers LARIS EA 7315, Image Signal et Sciences du Vivant Angers France

**Keywords:** virtual reality, neurosurgery, social cognition, awake surgery

## Abstract

**Background:**

In awake craniotomy, it is possible to temporarily inactivate regions of the brain using direct electrical stimulation, while the patient performs neuropsychological tasks. If the patient shows decreased performance in a given task, the neurosurgeon will not remove these regions, so as to maintain all brain functions.

**Objective:**

The objective of our study was to describe our experience of using a virtual reality (VR) social network during awake craniotomy and discuss its future applications for perioperative mapping of nonverbal language, empathy, and theory of mind.

**Methods:**

This was a single-center, prospective, unblinded trial. During wound closure, different VR experiences with a VR headset were proposed to the patient. This project sought to explore interactions with the neuropsychologist’s avatar in virtual locations using a VR social network as an available experience.

**Results:**

Three patients experienced VR. Despite some limitations due to patient positioning during the operation and the limitation of nonverbal cues inherent to the app, the neuropsychologist, as an avatar, could communicate with the patient and explore gesture communication while wearing a VR headset.

**Conclusions:**

With some improvements, VR social networks can be used in the near future to map social cognition during awake craniotomy.

**Trial Registration:**

ClinicalTrials.gov NCT03010943; https://clinicaltrials.gov/ct2/show/NCT03010943 (Archived at WebCite at http://www.webcitation.org/70CYDil0P)

## Introduction

Social cognition includes all complex cognitive processes involved in social interaction such as nonverbal language (facial and bodily nonverbal cues as affective prosody), empathy, and theory of mind (ToM). Following brain surgery, the impairment of nonverbal cue decoding, such as expression of facial emotions, eye gaze, body gestures, and prosody can lead to ToM deficits. Patients often experience difficulties with understanding humor and as well as conceptualizing and understanding thoughts and beliefs, emotions, feelings and desires, behaviors, actions, and intentions of other people. In recent times, these sequelae were largely misunderstood by neurosurgeons and compared with postsurgical impairment of language or executive functions, with few evaluations concerning social cognition having been published [1-6].

As previously done for language, it is now possible to propose a substrate for social cognition based on parallel and large-scale interactive distributed brain networks [7]. However, unlike language, this substrate cannot be reliably localized based on anatomical criteria alone, mostly due to the individual variations. Individual brain mapping by direct electrical stimulation (DES) during awake craniotomy is therefore essential. The procedure has been well documented [8]. Briefly, it is possible to temporarily inactivate regions of the brain using DES, while patients perform neuropsychological tasks. If a patient shows decreased performance in a given task, the neurosurgeon will not remove these regions, so as to maintain brain function.

Compared with motor or language mapping, nonverbal language mapping has not been performed yet. This is due to the difficulties involved in adapting classic bedside tasks to awake surgery conditions.

In 2014, we started to explore the use of virtual reality (VR) during awake craniotomy with patients wearing a virtual reality headset (VRH). We previously developed an app for VRH to explore visuospatial cognition [9]. We are now performing a larger study evaluating the tolerance and safety of VRH and 3D immersive experiences in patients undergoing awake craniotomy and brain mapping by DES. Herein, we describe a VR experience, the interaction with an avatar using a social VR platform, and highlight its advantages, limitations, and future applications for perioperative mapping of social cognition.

## Methods

This was a single-center, prospective, unblinded trial (ClinicalTrials.gov base identifier: NCT03010943), which was performed in compliance with all regulatory and ethical guidelines for clinical research. All patients signed a written informed consent.

The inclusion criterion was patients aged >18 years who were hospitalized for treatment of a tumor or any type of surgical lesion near the language region of the brain. The exclusion criteria were all contraindications to an awake surgery (cognitive impairment, aphasia, and morbid anxiety). The main objective was to assess procedural feasibility and safety.

This study was performed using a Samsung Gear VR combined with a Samsung S7 smartphone (android platform) and headphones. After general and local anesthesia, the patient was positioned lying on his side, with a rigid pin fixation of the head. Once the craniotomy was completed and the dura was opened, we awakened the patient. Electroencephalography signals were recorded using a subdural electrode. After the cortex was exposed, language mapping was performed by a neuropsychologist using an image denomination task on a digital tablet. The mapping took place as previously described [10,11]. DES was applied with a bipolar electrode delivering a biphasic current (60Hz, 1 ms pulse width, current amplitude ranging from 2 to 8 mA over 2-3 s).

To prevent interference with the routine procedure of awake craniotomy and language brain mapping, we decided to duplicate the image naming task viewed in VRH (two dimensions, 2D) and then in stereoscopy (three dimensions, 3D; an app based on Unity 3D software with an interface allowing VRH communication via a computer and Bluetooth connection). Further VR experiences with a relaxing film were proposed at the end of the tumor resection while the wound was being closed. These options included interaction with the neuropsychologist’s avatar in virtual locations; this option is the focus of this paper. For this experience, we used the vTime app, a social network in VR [12]. This app allows users to create an avatar and socialize with other people in virtual environments. The avatar can be piloted on a smartphone or in conjunction with a VRH using a game controller.

## Results

A total of 3 patients used the vTime app during wound closure (2 males and one female; mean age, 54 years). Only 1 participant had a previous VR experience. Before the surgery, all the patients were trained without any issues.

Patients used a standard avatar and an account opened by the Department of Neurosurgery to preserve anonymity. They interacted with an avatar piloted by the neuropsychologist, who also wore a VRH, under the control of a physician who participated in the meeting and controlled the scene on a smartphone connected to the app. This allowed continual monitoring of the operation (Multimedia Appendix 1). The mean time of connection and interaction was 10 minutes.

During DES of the left inferior frontal gyrus (pars opercularis), all patients failed to perform the 2D and 3D language and motor tasks. All deficits disappeared when DES was stopped, and the patients were allowed to recover. The stimulated areas were not resected. Patients were neurologically intact.

During the social cognition experiences, the patients passively viewed the neuropsychologist’s avatar and reproduced and commented on his gestures. Alternatively, they assumed more active roles, controlling their own avatars with a game controller in their hands (Multimedia Appendix 2).

Despite the discomfort associated with the awake surgery environment and other tasks completed with VRH, no patient experienced eye strain, nausea, or any sign of “VR disease.” No seizures occurred while the patients looked at the VR experience.

## Discussion

### Principal Findings

As described previously, social cognition includes nonverbal language, empathy, and ToM. These functions are explored at the bedside by complex neuropsychological tasks batteries including story movies, comic strips, or interactive games that depict a short story. These tasks require time to be performed, meaning they are not compatible with the brain mapping conditions (DES length inferior to 4 seconds, fast response, and no ambiguity in the answer).

VR approaches that allow interactions with an avatar are commonly used in cognitive neurosciences [13]. There is consistent evidence that avatars are perceived in a similar manner to real human beings and can be used to explore the complex processes of nonverbal language, empathy, and ToM [14]. In VR, the social interactions are governed by the same social norms as social interactions in the real world (for example social norms related to gender, interpersonal distance, and eye gaze) [15].

VR can imitate complex social situations, even for the patients undergoing awake craniotomies. The potential of VR lies in its increased real-life environment validity compared with screen-based studies. Rather than being a passive observer of stimuli on a computer screen, participants in virtual environments become part of the depicted scene. Although an increase in ecological validity often results in a decrease in experimental control, immersive VR has the potential to combine the naturalness of everyday interactions with experimental controls required during brain mapping procedures.

Instead of developing a specific app to test and map social cognition during awake brain surgery, we decided to test the potential of the available VR social networks. Several VR social platforms are already available, such as vTime [12], Oculus, Facebook Spaces, PLUTOVR, and AltspaceVR. Interestingly, these platforms take different approaches for conveying nonverbal language: arms, hands, head, and mouth movement and gaze.

For our trial, we chose Samsung VR, a low-cost, high-quality, customizable wireless device, with an optional pad control and a game controller. The VR social network vTime is compatible with the Samsung VRH [12]. The vTime app allows interaction with several avatars and positional control in different virtual environments. The avatar can point anywhere within the scene and produce gestural expressions such as OK, Thumbs Up, Clap, Thumbs Down, Blow Kiss, etc [12]. It is also possible for the user to touch other avatars or to take control of his or her personal space.

We demonstrated that patients undergoing awake craniotomies can wear a VRH and interact with an avatar piloted by a neuropsychologist.

### Limitations

We experienced some difficulties and limitations using vTime [12]. During an awake surgery, the patient is usually lying on his or her back or side with the head immobilized using a Mayfield skull clamp. The vTime app [12], as with most VR social experiences, is not well-adapted for use in this position and cannot make use of the 360 degree view. Further, there is no option to control the orientation of the virtual environment. In our experience, vTime can only be used with side-lying patients. Restrictions of neck, limbs, and face movements can affect psychological testing. If the patient does not lie on his or her side to visualize the neuropsychologist avatar, he or she cannot be tested. Despite this limitation, all awake surgeries were performed in our institution with the patient lying on his or her side. Mobility restrictions limit the patient’s ability to explore the 360-degree environment, potentially limiting the use of vTime [12] to explore visuospatial cognition. Moreover, it is not possible to control facial expression and eye gaze, which are potent nonverbal language cues. An app dedicated to awake surgery is currently in development to overcome this limitation.

### Conclusions

We showed that it is possible to use a VR social network during awake craniotomy and to test gesture communication. Progress in VR development is currently promising, and some VRHs even allow facial expressions to be captured and transferred to a virtual avatar in real time, opening a new level of virtual human interaction. We are convinced that these improvements could be applied to further research for awake craniotomy, nonverbal language, empathy, and ToM in the near future.

## Appendix

Multimedia Appendix 1 During awake brain surgery, the patient and the neuropsychologist (A) performing a language task; (B) Direct electrical stimulation and mapping of the cortex during the task; and (C) and (D) the patient and the neuropsychologist communicating with the VR social network.

Multimedia Appendix 2 Video showing interaction between the patients and the neuropsychologist avatar using Vtime [12] in order to test social cognition during awake brain surgery.

## Abbreviations

DES: direct electrical stimulation
RCT: randomized controlled trial
ToM: theory of mind
VR: virtual reality
VRH: virtual reality headset
