# Supplementary figures and images for: Using a Virtual Reality Social Network During Awake Craniotomy to Map Social Cognition: Prospective Trial
Source: J Med Internet Res. 2018 Jun 26;20(6):e10332. doi: 10.2196/10332 (PMC6039768; doi:10.2196/10332)

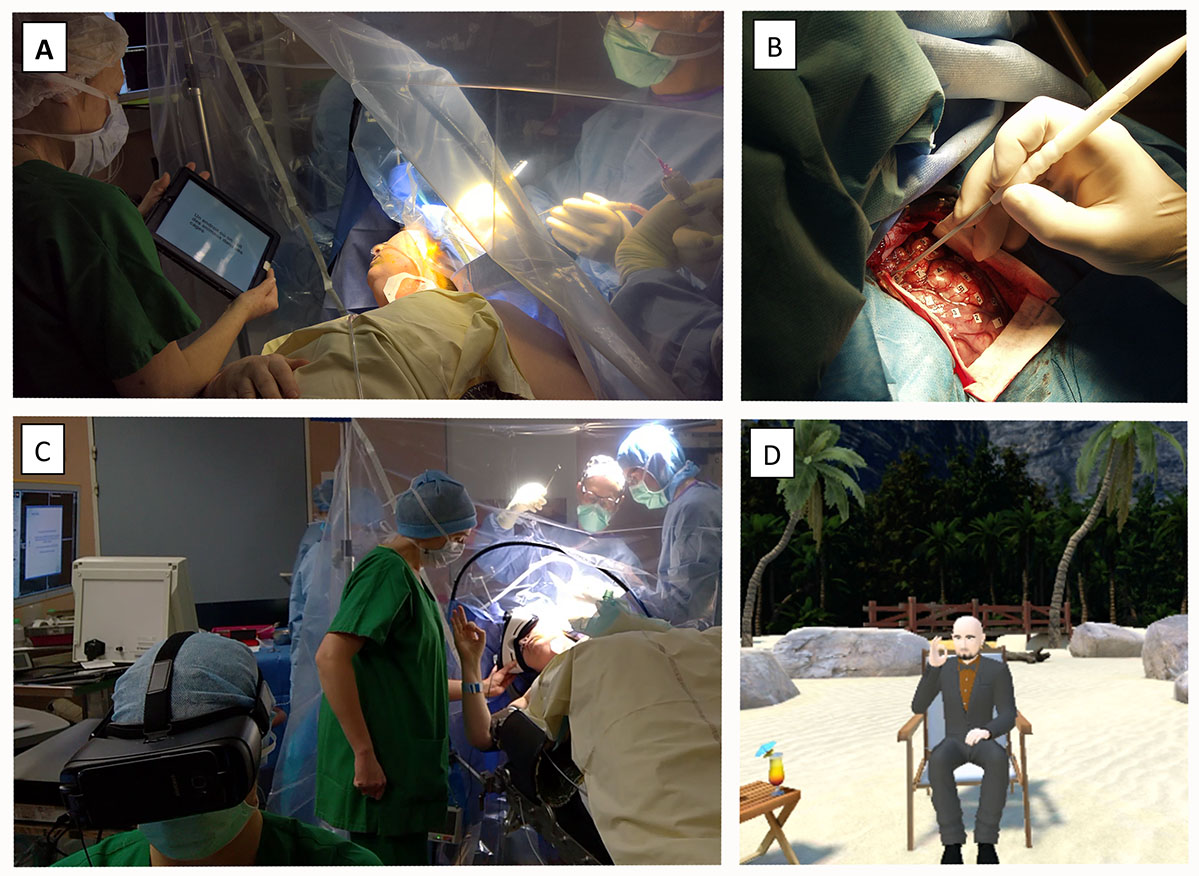

Supplement: Multimedia Appendix 1 [file jmir_v20i6e10332_app1.png]
